# When is fire weather extreme enough for active fire spread in Canada?

**DOI:** 10.1098/rstb.2023.0465

**Published:** 2025-04-17

**Authors:** Xianli Wang, Tom Swystun, Jacqueline Oliver, Kathryn Levesque, Mike D. Flannigan

**Affiliations:** ^1^ Northern Forestry Centre, Canadian Forest Service, Natural Resources Canada, Edmonton, Alberta T6H 3S5, Canada; ^2^ Great Lakes Forestry Centre, Canadian Forest Service, Natural Resources Canada, Sault Ste. Marie, Ontario P6A 2E5, Canada; ^3^ Department of Natural Resource Science, Faculty of Science, Thompson Rivers University, Kamloops, British Columbia V2C 0C8, Canada

**Keywords:** spread days, fire weather, thresholds, Canada

## Abstract

A spread day is defined as a day in which fires grow by a substantial amount of area, usually during high or extreme fire weather conditions. Accurately identifying a spread day under various environmental conditions could help both our understanding of fire regimes and with forecasting and managing fires on the ground. Although spread days could occur within a spectrum of fire weather conditions, a threshold is important to fire management and fire research. This study explores the relationships between spread days and fire activity in the forested area of Canada by spatially and temporally matching daily fire growth to interpolated daily gridded fire weather between 2001 and 2021. Using accumulative area burned density functions, we identified the fire weather conditions for spread days by Canadian Ecozones both annually and seasonally. Using these identifiers as thresholds, we estimated how extreme fire weather needs to be for a spread day to occur, and the proportions of potential spread days (PSDs) that would most likely be realized in real fire spread at various Canadian Ecozones. Our results showed that the median-level fire-conducive weather conditions are sufficient to support active fire growth, and on average, about 22–30% of such days may be realized in real fire spread at various Canadian Ecozones.

This article is part of the theme issue ‘Novel fire regimes under climate changes and human influences: impacts, ecosystem responses and feedbacks’.

## 1. Introduction

Making connections between fire weather (e.g. temperature, precipitation, humidity and wind speed) and fire activity (e.g. area burned, size and frequency of fires) has been a long-lasting effort in fire research over the past century. One of the major challenges is that these relationships are mostly nonlinear and multivariate at best (e.g. [1]). To compensate, various fire weather index (FWI) systems have been constructed, including the Canadian FWI System [2] and the National Fire Danger Rating System [3]; in the US, the McArthur Forest Fire Danger Index ([4,5] in Australia, and the Haines index (C-Haines; [6,7]), which attempts to characterize atmospheric humidity and vertical stability. In addition, the hot–dry–windy index [8,9] and vapour pressure deficit [10,11] have been promoted in recent years. However, even with all these old and new indices, a direct statistical or mathematical relationship between a specific fire weather measurement and fire activity is rarely found (but see [12]).

In recent years, researchers have found some strong linkages between fire activity and extreme fire weather conditions based on a simple concept, namely the ‘spread day’ (e.g. [13,14]). Fires burn most of their areas within a few days of extreme fire weather conditions [15], regardless of how long the actual fire durations were, and such days are called the spread days [13]. A power law relationship exists between fire duration and size only when the duration is measured as the number of spread days [16], and the potential maximum number of spread days also shows the prediction power of annual area burned and fire frequency [17,18]. However, there is still no common standard for how to identify a spread day during the lifetime of a fire. Two aspects of identifying a spread day are critical to both academic research and fire management practice: one is the actual fire growth and the second is the fire weather thresholds to separate a spread day from a non-spread day (e.g. [14]). An actual spread day could be considered the realization of the fire weather potential.

Identification of an actual spread day is mostly arbitrary, and a threshold rate of spread (ROS) value is normally used to determine whether one day is a spread day during a fire (e.g. [14,19]). Consideration for a spread day is a two-step procedure, where step one is the daily fire growth delineation and step two is deciding the speed of fire spread, namely the ROS, which is normally generated based on a circular model [14] as a threshold for a spread day. An often-used daily fire growth delineation algorithm was developed by Parks [20] based on the final fire perimeter and daily remote-sensing hotspots within that perimeter. Other methods may be more accurate in delineation but require higher-resolution observations (e.g. [21–23]), which are not always available at large spatial and temporal scales. These ROS thresholds are usually chosen based on trial and experience. A robust procedure for identifying such thresholds has yet to be developed (e.g. [13,14]). These challenges exist because both fire perimeters and hotspots are not exempt from errors, and the ROS of a fire is difficult to accurately quantify (e.g. [24]).

Similarly, for fire weather conditions that may trigger and sustain a spread day, there is no clear-cut threshold of any metrics to simply separate a spread day and a non-spread day, especially when the definition of a spread day on the ground remains debatable. In fact, a spread day may occur at a gradient of specific FWI values (e.g. the fine fuel moisture code (FFMC) from FWI System), although the probability of its occurrence varies (e.g. [25,13]). A combination of the fire weather measurements may qualify as a threshold, but no research has been done to prove it. However, such thresholds or critical values are still desirable to fire management for preparedness, particularly when derived from a set of daily fire weather conditions. One inference was to use a probabilistic model to decide whether a spread day may occur or not, and if there is a 50% chance that a spread day may occur, those fire weather conditions are considered the threshold for a spread day [13,25]. However, such a technique relies on pre-defined ground truth spread days, which is not universally agreed upon as previously discussed.

A recent study [25] was able to identify the daily fire weather conditions that are responsible for the highest area burned during a fire season over a region, which stated that it is possible to identify a spread day fire weather threshold without predefining an actual spread day. Because the relationship between an FWI System variable and area burned roughly follows a bell-shaped curve [25], a more accurate identification would be the FWI System variable values where the accumulative area burned is 50%, assuming a perfect bell-shaped curve relationship exists. In this study, we identify the Canadian FWI System variable values corresponding to the daily fire growth of each individual fire that accounts for half of the area burned as the fire weather thresholds for a spread day. Using these threshold values, we explore (i) how extreme (in percentiles) the fire weather conditions need to be for a spread day to occur and sustain and (ii) the proportion of potential fire weather that is most likely to result in fire spread in Canada.

## 2. Method

### (a) Study area

**Figure 1.**
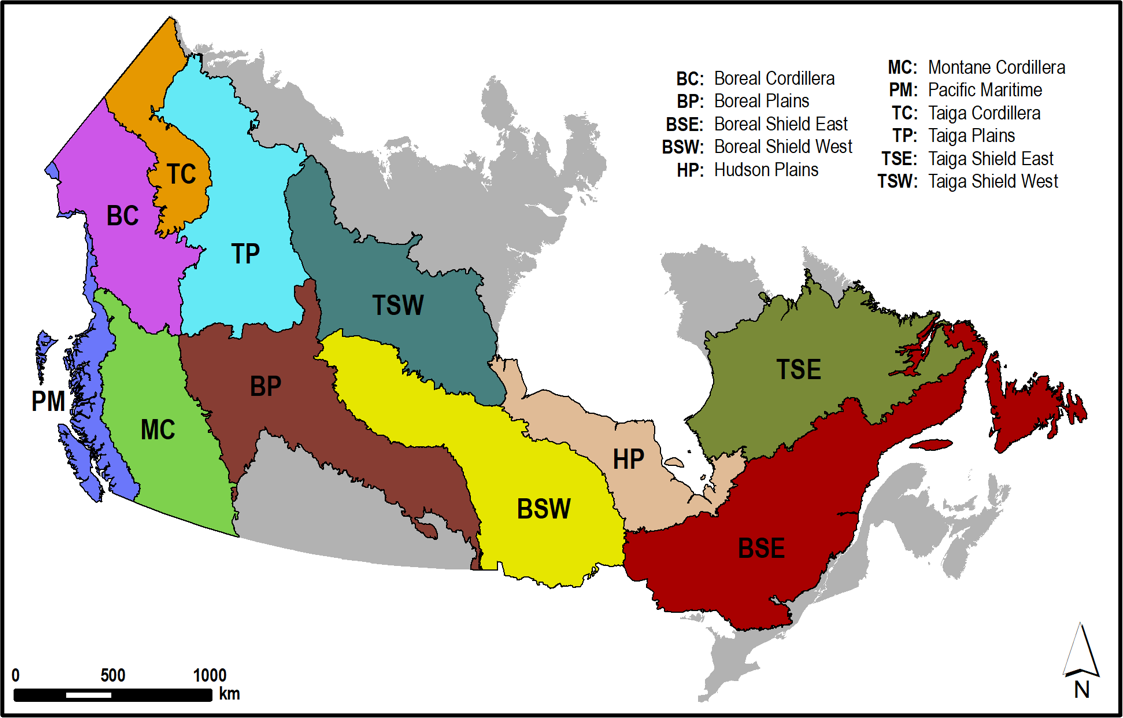
The Canadian ecozones.

Our study area is the forested continental landmass of Canada, where the Rocky Mountain range extends from British Columbia to western Alberta, northward into the Yukon, and includes parts of the Northwest Territories. The remainder of the study area, between the east of the Rocky Mountains and the Atlantic Ocean, is relatively flat. Our study area is dominated by three major biomes including the boreal forests in central Canada and bordered by the southern edge of the Tundra, the temperate coniferous forests on the west coast and the temperate broadleaf and mixed forests that cover the east coast and the Great Lakes area. We used the Canadian Ecozones [26] as the analysis unit but we split the Taiga Shield and Boreal Shield Ecozones into west and east components owing to climate and fire activity disparities, following ([27]; figure 1).

### (b) Data

#### (i) Fire weather index system

The Canadian Forest FWI System [2] has been used to assess relative fire danger in Canada and many other countries (e.g. France, Portugal, Spain, Indonesia, Malaysia, Mexico, New Zealand and the USA). Based on the screen-level air temperature and relative humidity, 10 m open wind speed and 24 h accumulated precipitation collected at local noon, the FWI System calculates six indices to characterize potential fuel moisture and fire behaviour [2]. These indices include the FFMC, the Duff Moisture Code (DMC) and the Drought Code (DC), which track moisture content of surface, duff and deep fuel layers, respectively. The Initial Spread Index (ISI), the Build-up Index (BUI) and the FWI, on the other hand, assess potential fire spread, fuel availability and fire intensity, respectively. A higher FWI System moisture code indicates drier conditions, and a higher index unit represents the potential for more extreme fire behaviour owing to more severe underlying fire weather conditions.

#### (ii) Fire data

The National Burned Area Composite (NBAC) dataset [28] was used in this study. The NBAC dataset is a fire polygon database derived from 30 m Landsat imagery and high-quality agency imagery that includes fires that occurred between 1986 and 2021. The dataset is considered the most consistent and accurate wildfire polygon database in Canada. In this study, fires larger than 50 ha (e.g. [17]) between 2001 and 2021 were used, in accordance with the availability of Moderate Resolution Imaging Spectroradiometer (MODIS) and Visible Infrared Imaging Radiometer Suite (VIIRS) products (https://firms.modaps.eosdis.nasa.gov/).

#### (iii) Fire weather data

A subset of the 0.25° resolution gridded Global Fire Weather Indices dataset [29] was used in our study. This database took the European Centre for Medium-range Weather Forecasts ERA5-HRS Reanalysis product [30] as inputs and calculated the FWI System indices using R functions in the *cffdrs* package [31]. We also obtained the updated ERA5-based fire weather database for the years 2019−2021 from the authors [29], which enabled us to include fire occurrence as part of our analysis.

#### (iv) Mapping daily fire growth

A fire growth mapping technique developed by Parks [20] was used to delineate daily fire growth for all fires considered in this study. We mapped daily fire spread using the NBAC fire perimeters (≥50 ha) and MODIS (https://firms.modaps.eosdis.nasa.gov/usfs/) hotspots for fires that occurred from 2001 to 2012 and used MODIS hotspots and VIIRS hotspots for fires that occurred from 2013 to 2021. Using the NBAC fire perimeters to constrain the final fire size of the interpolation, daily fire growth was mapped at a 30 m resolution by spatially interpolating the 1 km (MODIS) or 375 m (VIIRS) resolution remote-sensing hotspots [20].

#### (v) Pairing daily fire growth with fire weather condition

To correspond the daily fire growth with the daily fire weather condition for all fires within the study region from 2001 to 2021, the nearest ERA5-based fire weather grid points [29] within the daily burned area were identified. To increase accuracy, a regression Kriging method using elevation as a covariate (e.g. [32,33]) was used to interpolate the ERA5 FWI System variables to 100 m resolution grid points within the daily fire growth polygon. Each of the spatial interpolation models was built based on ERA5 grid points within a 50 km buffer around each fire growth event; however, if the variogram failed owing to a lack of points, we extended the buffer to 200 km. The variogram was fitted using the ‘autofitVariogram’ function, and the regression Kriging model was carried out with the ‘krige’ function from the R-packages ‘*automap*’ [34] and ‘*gstat*’ [35,36], respectively. A mean value was found for each of the FWI System variables, except where an FWI median value was calculated owing to its exponential relationship with potential fire intensity [2]. It is with these interpolated points that daily fire growth and fire weather conditions were paired.

### (c) Analysis

#### (i) FWI System variable thresholds of a spread day

In order to identify the FWI System variable thresholds for a spread day, we randomly selected 10 of the 21 available years of the daily fire growth paired with fire weather data. We fitted a generalized additive model (GAM [37]) between the accumulated area burned along each of the FWI System variables using the gam function from the R package ‘mgcv’ [38]. From this regression model, we identified the FWI System index values corresponding to 50% of the area burned. We repeated this process 100 times, and we then calculated the mean and standard deviation of each FWI System variable value. The same analysis was performed based on fires over the whole study area, as well as by Ecozone and season (i.e. spring (March, April and May—MAM), summer (June, July and August—JJA) and fall (September, October and November—SON)) with sufficient fire records.

#### (ii) Relationship between fire weather condition and area burned

To understand the level of burning conditions that may trigger a spread day, we identified the percentiles corresponding to the FWI System variable values below which 50% of the total area was burned. This was done for the whole study area, as well as by Ecozone and season, using the paired daily fire growth and fire weather condition data. We chose Ecozone as the analysis unit because it has been widely used in fire research in Canada (e.g. [13,16,25,39]) and because of the limited samples we could obtain to perform reliable statistical analysis.

To better understand the relationship between the fire weather conditions and fire activity, we calculated the percentage of area burned above various FWI System variable percentiles (between 50 and 99 with 5% increment, except for the last step between 95 and 99%) by Ecozone and season, as well as for the whole study area. These values were obtained through a simulation procedure where 10 out of 21 years of daily fire growth and weather were repeatedly (*n* = 100) and randomly selected. With each iteration, a nonlinear regression model (GAM) was fitted between the accumulated area burned along each of the FWI System variables based on the sampled data, and the area burned above these FWI System variable percentile thresholds was calculated accordingly.

#### (iii) Proportion of potential spread days being realized

With the selected ERA5-based fire weather grid points paired with each individual fire, we calculated the annual number of spread days (PSDs) for the time between 2001 and 2021, based on the thresholds we identified previously. Similarly, we calculated the number of spread days (realized spread days, RSDs) when fires occurred and spread for each individual fire. The RSD/PSD ratios for each individual fire were summarized by Ecozone, season and the whole study area. These ratios are an indication of how much fire weather potential could be used after a fire is ignited.

## 3. Results

### (a) Fire weather index system variable thresholds of a spread day

**Figure 2.**
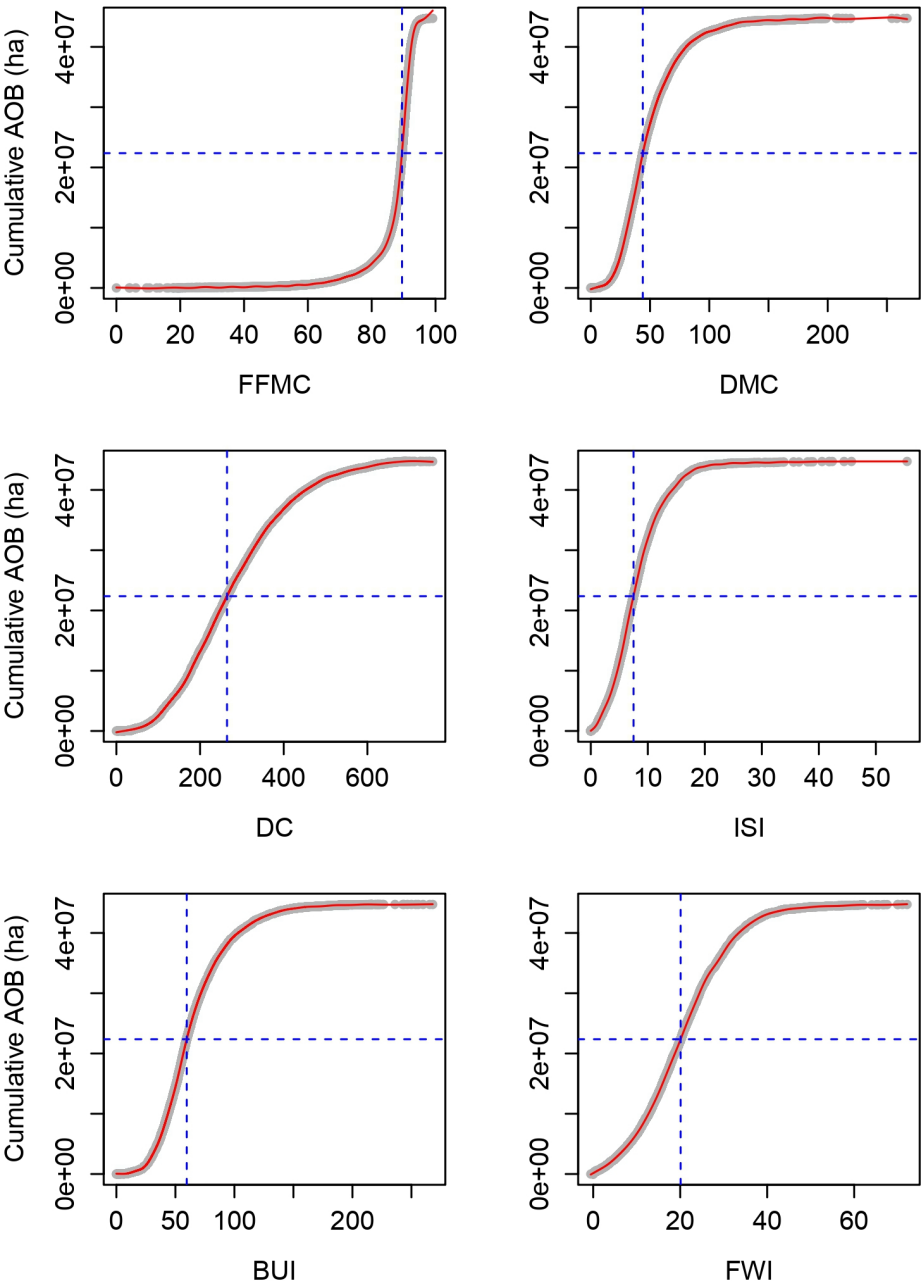
FWI System variable thresholds where 50% of the annual area burned (AOB) occured at the national level, the intersection between the blue dashed lines on the accumulated area burn curve fitted with a GAM along FWI System variables.

FWI System variable thresholds for a spread day to occur (figure 2) based on the fires observed over the study area showed that more severe fire weather conditions are needed in the spring and less in the fall (table 1). We also found that the summer thresholds are similar to those for all seasons combined. Similar patterns are also observed in some of the Ecozones (table 2 and electronic supplementary material, table S1[40]), where the results of the shoulder seasons (i.e. spring and fall) of a few Ecozones (e.g. Taiga Cordillera (TC), Pacific Maritime (PM) and Taiga Shield East (TSE)) are not reliable owing to the small sample size available (electronic supplementary material, table S1). Among all six FWI System variables considered, FFMC varies the least among all Ecozones (between 86.9 and 90.8) and DC showed the largest variations (between 119.2 and 442.4). As expected, the averaged values among the Ecozones (table 2, electronic supplementary material, table S1) are similar to those obtained based on data of the whole country (table 1).

**Table 1.** Mean FWI System variable values (s.d.) below which 50% of the total area was burned for the whole study area. These values were derived through a simulation by repeatedly (*n* = 100) randomly selecting 10 out of the 21 available years of daily fire growth and fire weather data between 2001 and 2021 and by fitting a nonlinear regression model between the accumulated area burned along each of the FWI System variables. BUI, build-up index; DC, drought code; DMC, Duff moisture code; FFMC, fine fuel moisture code; FWI, fire weather index; ISI, initial spread index.`

indices	all year	spring	summer	fall
FFMC	89.4 (0.2)	90.8 (0.7)	89.2 (0.2)	88.6 (0.3)
DMC	44.0 (1.6)	45.1 (5.9)	44.1 (1.8)	36.5 (3.0)
DC	262.8 (20.0)	147.5 (26.1)	271 (19.2)	418.7 (40.9)
ISI	7.5 (0.3)	9.1 (0.8)	7.4 (0.3)	7.2 (0.6)
BUI	59.8 (2.4)	51.5 (6.8)	60.3 (2.8)	58.9 (4)
FWI	20.1 (0.5)	22.0 (2.2)	19.9 (0.6)	19.2 (1.2)

**Table 2.** FWI System variable values below which 50% of the annual total area was burned by Canadian Ecozone (see also captions in table 1). BUI, build-up index; DC, drought code; DMC, Duff moisture code; FFMC, fine fuel moisture code; FWI, fire weather index; ISI, initial spread index.

ecozone	FFMC	DMC	DC	ISI	BUI	FWI
Taiga Plains (TP)	88.8 (0.3)	48.3 (5.4)	366.1 (41.8)	6.5 (0.3)	68.4 (7.9)	19.2 (1.4)
Taiga Shield West (TSW)	89.1 (0.4)	47.8 (6.6)	302.2 (27.2)	7.8 (0.5)	66.5 (7.5)	21.1 (1.6)
Boreal Shield West (BSW)	89.3 (0.3)	41.5 (1.5)	255.5 (12.8)	7.4 (0.4)	58.0 (2.2)	19.3 (0.8)
Boreal Plains (BP)	90.1 (0.4)	47.0 (2.6)	256.3 (36.7)	8.0 (0.4)	62.7 (3.9)	22.0 (1.1)
Taiga Cordillera (TC)	86.9 (1.3)	43.7 (5.8)	238.2 (49.5)	4.7 (0.6)	53.0 (8.2)	12.9 (1.9)
Boreal Cordillera (BC)	88.1 (0.5)	38.7 (3.5)	274.6 (33.1)	5.5 (0.6)	55.9 (4.9)	14.7 (1.4)
Pacific Maritme (PM)	88.3 (1.1)	41.7 (5.2)	286.3 (33.8)	4.9 (0.6)	60.5 (7.0)	13.9 (2.6)
Montane Cordillera (MC)	90.8 (0.7)	75.3 (7.6)	442.4 (27.4)	7.6 (0.7)	106.2 (10.2)	25.3 (3.0)
Hudson Plains (HP)	89.1 (0.9)	37.6 (5.0)	227.9 (46.0)	8.5 (1.5)	50.9 (4.9)	19.5 (3.4)
Boreal Shield East (BSE)	90.2 (0.3)	39.0 (2.5)	147.7 (18.8)	10.1 (0.3)	46.7 (3.2)	21.7 (1.0)
Taiga Shield East (TSE)	89.3 (0.4)	28.8 (3.0)	119.2 (13.0)	9.3 (1.4)	35.6 (3.2)	17.7 (1.6)
mean (s.d.)	89.1 (0.6)	44.5 (4.4)	265.1 (30.9)	7.3 (0.7)	60.4 (5.7)	18.8 (1.8)

### (b) Relationship between fire weather condition and area burned

The corresponding percentiles of these FWI System variable thresholds for a spread day are relatively high across the entire study area (table 3), with the exception of DC. The fast-reacting FWI System variables (i.e. FFMC, ISI and FWI) are higher than the 60th percentile overall and by season except for FFMC in the fall, which is close to the 60th percentile (59.4). The slow-reacting FWI System variables (i.e. DMC, DC and BUI) are a bit lower—less than the 60th percentile except for the spring. However, these patterns do not hold true by Ecozones (table 4 and electronic supplementary material, table S2), where they are mostly above the 50th percentiles, indicating that active fire growth may occur as soon as fire weather conditions are above the median.

**Table 3.** Percentiles corresponding to the FWI System variable values below which 50% of the total area burned for the whole study area. BUI, build-up index; DC, drought code; DMC, Duff moisture code; FFMC, fine fuel moisture code; FWI, fire weather index; ISI, initial spread index.

indices	all year	spring	summer	fall
FFMC	64.5	62.7	62.6	59.4
DMC	59.1	66.7	58.8	53.1
DC	42.7	73.5	45.0	53.1
ISI	68.7	62.9	68.6	63.1
BUI	56.3	69.2	56.2	52.7
FWI	68.1	70.2	67.7	62.3

**Table 4.** Percentiles corresponding to the FWI System variable values below which 50% of the annual total area burned by Ecozone. BUI, build-up index; DC, drought code; DMC, Duff moisture code; FFMC, fine fuel moisture code; FWI, fire weather index; ISI, initial spread index.

ecozone	FFMC	DMC	DC	ISI	BUI	FWI
Taiga Plains (TP)	63.7	65.5	63.5	67.3	64.2	70.2
Taiga Shield West (TSW)	62.8	64.4	52.9	68.1	63.8	69.7
Boreal Shield West (BSW)	67.6	66.0	46.9	68.7	65.0	71.2
Boreal Plains (BP)	65.1	60.6	38.2	66.3	54.5	68.4
Taiga Cordillera (TC)	58.4	64.1	50.8	61.6	57.4	63.2
Boreal Cordillera (BC)	63.3	58.4	50.7	69.3	58.6	68.0
Pacific Maritime (PM)	57.3	53.9	51.5	55.3	53.6	53.2
Montane Cordillera (MC)	52.7	58.8	64.0	56.6	62.3	58.2
Hudson Plains (HP)	67.5	68.3	44.0	69.6	62.7	68.7
Boreal Shield East (BSE)	64.4	65.8	58.2	70.0	68.7	73.1
Taiga Shield East (TSE)	68.9	57.1	45.2	72.5	54.3	70.7
mean (s.d.)	62.9 (4.7)	62.1 (4.3)	51.4 (7.6)	65.9 (5.4)	60.5 (4.8)	66.8 (5.8)

**Figure 3.**
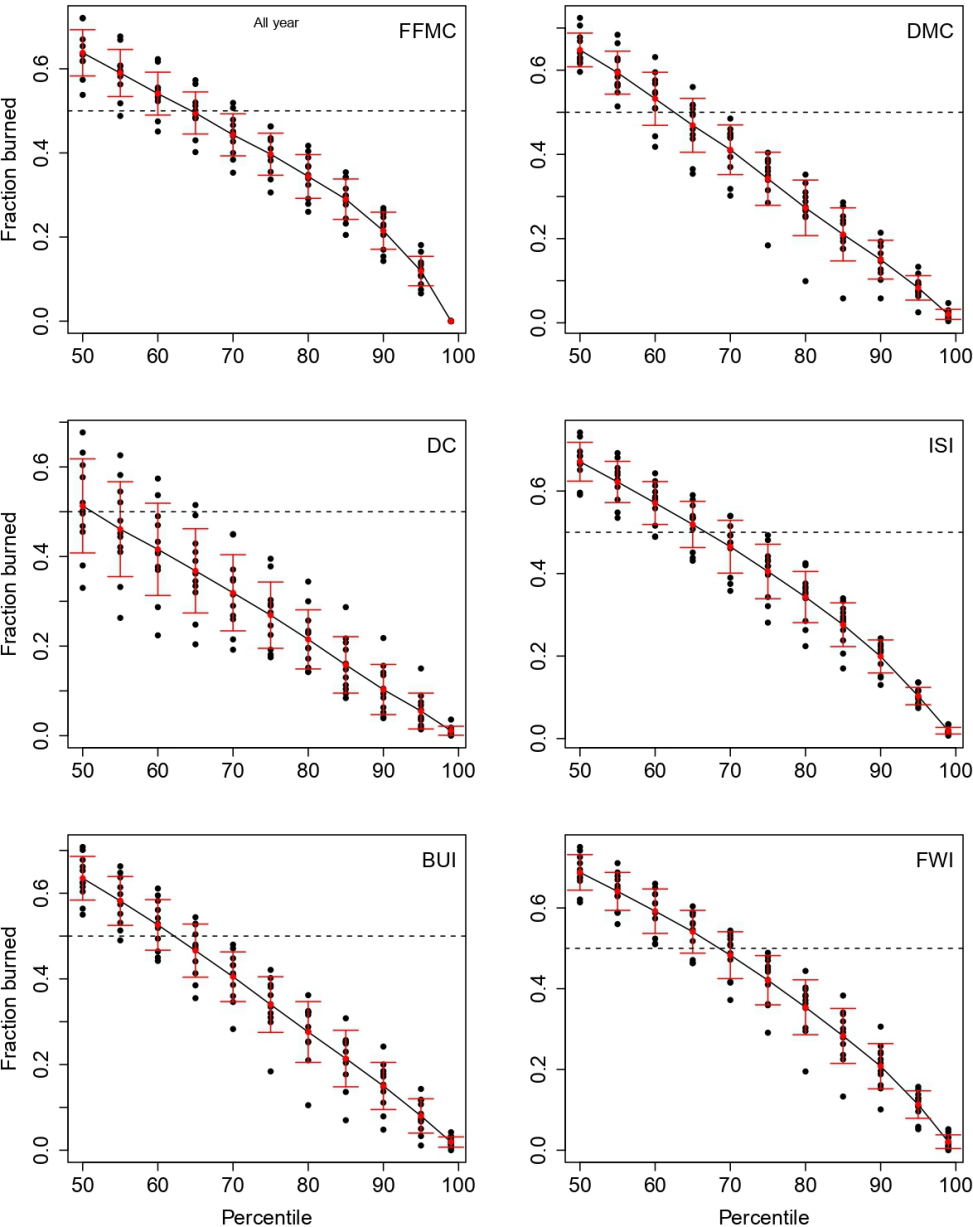
Proportion of area burned (annual) at various FWI System variable percentiles by ecozone. Red dots and lines are mean and standard deviation among the ecozones considered in the study. The horizontal dashed lines represent when 50% of the area has burned.

For area burned at various FWI System variable percentiles (electronic supplementary material, table S3), we found that more than 60% of the area burned when the fire weather condition was above the median in most of the Ecozones (figure 3). The area burned under extreme fire weather conditions (e.g. 90th percentile) usually accounts for a relatively large portion of the area burned (around 20% for the 90th percentile; see figure 3 and electronic supplementary material, table S4).

### (c) Proportion of potential spread days realized into area burned

**Figure 4.**
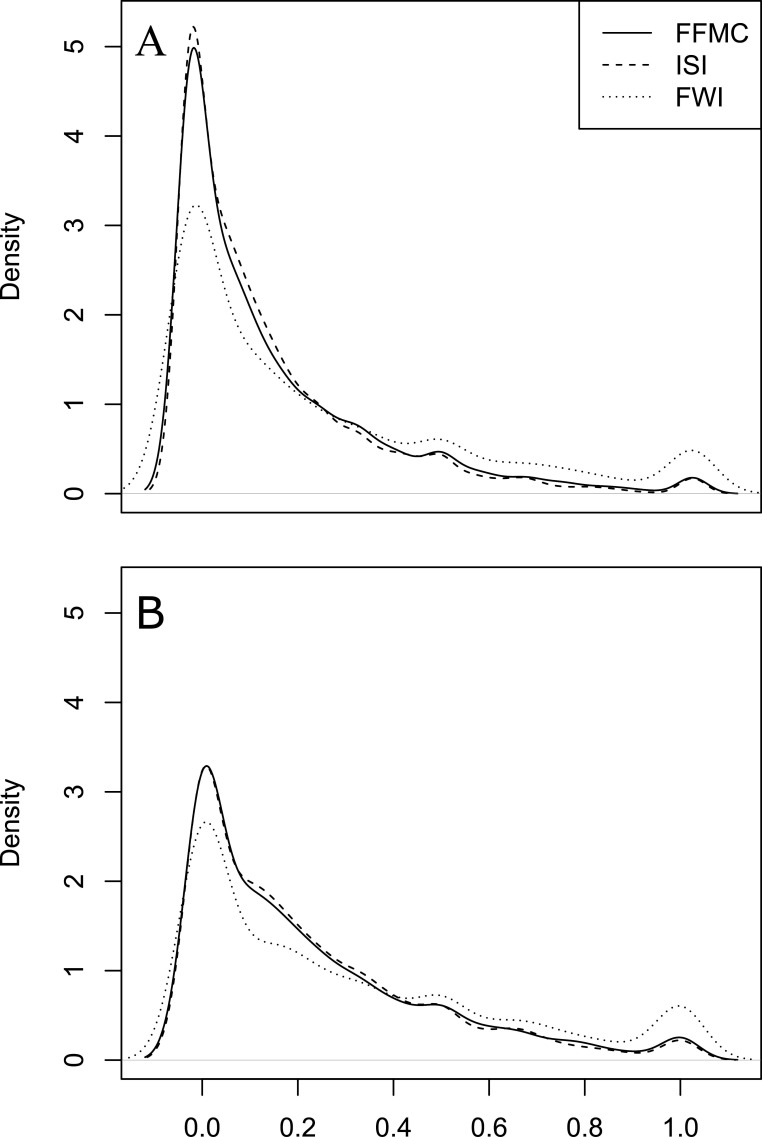
Density curves of RSD/PSD ratio by national thresholds (A) and Ecozone thresholds (B).

The expected proportion of PSDs being realized with given fires varies widely (table 5; figure 4). On average, based on the national thresholds of three fast-reacting FWI System variables (table 1), we see that 16.9–27.4% of the potential fire weather conditions are used when fires were ignited, and by Ecozone thresholds (table 2), these values vary between 22.6 and 30.4%. Ecozones in the Rocky Mountains (e.g. TC, BC, PM and MC) showed above-average RSD/PSD ratios in most of the cases (≥2/3 of both national and Ecozone level thresholds) and partially in BSW (1/2), TSW (1/3) and TSE (1/6; table 5). We also found that with an increase in the number of PSDs, there is also the possibility for these PSDs to be fully used by fires (i.e. RSD/PSD = 1.0; see electronic supplementary material, table S5, figures S1, S2 and S4).

**Table 5.** Proportional (%) potential spread days being used by fires given that a fire occurs at a specific location by ecozone. Three FWI System variables were selected to make the calculation based on the national thresholds (table 1) and the thresholds by Ecozone (table 2). FFMC, fine fuel moisture code; FWI, fire weather index; ISI, initial spread index.

ecozone	national thresholds			ecozone thresholds		
	FFMC	ISI	FWI	FFMC	ISI	FWI
TP	14.4 (19.6)	13.9 (19.6)	23.8 (30.2)	22.2 (24.0)	21.2 (23.7)	28.8 (31.8)
TSW	19.3 (22.5)	17.4 (18.8)	26.9 (28.9)	22.0 (23.7)	20.4 (21.7)	28.4 (30.6)
BSW	14.6 (17.9)	13.2 (15.3)	23.3 (27.9)	21.0 (23.2)	20.1 (21.4)	27.7 (29.5)
BP	12.9 (18.7)	11.5 (16.5)	16.4 (22.8)	21.6 (25.7)	21.2 (23.2)	25.0 (28.7)
TC	26.2 (32.9)	23.1 (33.4)	46.0 (39.9)	28.8 (28.8)	27.9 (29.4)	36.8 (36.1)
BC	21.9 (25.7)	21.4 (27.9)	40.2 (38.8)	31.7 (27.4)	32.1 (28.0)	42.1 (34.2)
PM	21.3 (30.5)	23.8 (39.9)	27.9 (40.0)	21.1 (30.1)	21.6 (27.0)	32.0 (33.8)
MC	17.5 (20.4)	19.7 (23.0)	26.0 (28.3)	24.5 (26.7)	24.3 (26.3)	32.7 (32.8)
HP	14.6 (18.5)	14.0 (15.3)	21.3 (25.2)	17.8 (22.4)	19.2 (22.4)	23.8 (27.2)
BSE	16.4 (21.0)	14.4 (16.3)	28.0 (30.5)	19.5 (25.0)	24.5 (27.5)	35.4 (34.2)
TSE	18.8 (25.4)	14.0 (16.3)	21.4 (28.6)	20.7 (25.4)	16.0 (21.5)	21.8 (26.3)
mean	18.0 (23.0)	16.9 (22.0)	27.4 (31.0)	22.8 (25.7)	22.6 (24.7)	30.4 (31.4)

## 4. Discussion

The identification of a spread day when a fire is ignited is crucial to both fire suppression and firefighter safety. From the perspective of fire suppression, identifying spread days after a fire event is far less important than predicting such a day in advance. The FWI System variable conditions for a spread day are therefore desirable and useful because the same index system is widely used in fire management and operation [41]. This study fitted a nonlinear regression model between the accumulated area burned along each of the FWI System variables. The fitted curve was used to identify the FWI System index values corresponding to 50% of area burned as the spread day identifiers because such a value normally corresponds to the most area burned [25]. Based on these critical values, we further explored the relationship between fire-conducive weather and the area burned under such conditions and found that a spread day may occur right after the fire weather condition above the historical average (median). In addition, we found that although on average about 20% of the fire weather potentials were used by a given fire occurrence, it is still possible that all those spread days could be used by fires.

We acknowledge that the new threshold values found in this study are similar to those found in [25]; however, we believe that the new results are theoretically more accurate. The results based on the interpolated finer resolution FWI System variable values used in this study did not show changes in the spatial variation patterns over the whole study area that were shown in [25]. This may imply that better fire weather products (in terms of reflecting local weather conditions) and more accurate fire growth delineation methods are needed for more accurate estimates of the crucial fire weather conditions that may support a spread day (see also [25]). In addition, with more fire observations in fire-scarce areas (e.g. PM), our understanding of the fire and weather relationship would be improved. However, these are beyond the scope of this study and will have to be improved in the future.

Our results have demonstrated that the fire weather conditions do not have to be very extreme for a spread day to occur. As previously discovered, fire spread is normally driven by the fast-reacting fire weather conditions (e.g. [13,25]), and that is the reason—even in early spring while fuel availability is limited—why fires still spread quickly. This was demonstrated in western Canada’s 2023 fire season. Extreme fire weather conditions may be responsible for a smaller proportion of area burned, e.g. 20% of area burned occurred within the highest 10% of the extreme fire weather conditions, owing to the low frequency of such fire weather conditions. However, when compared with a uniform distribution, the proportion of area burned is already double the proportion of the fire weather conditions. It is therefore easy to suspect that a higher proportion of area will be burned when such extreme conditions become more frequent in the future (e.g. [1,18,39]).

For a PSD to become a real fire spread at any location, ignition must first occur. Because not all PSDs are temporally continuous, normally only a portion of the fire weather potentials are used within a specific fire. On average, we found that about 17.0–30.0% of the PSDs are used given fires that occurred on the ground. However, there are also cases where all PSDs were used by a fire, indicating that in such cases, all PSDs are distributed within one long fire-conducive weather spell, and that fires were ignited as soon as the fire-conducive weather presented. With an increase in the length of such an event (e.g. [18]), the number of PSDs being used by fire would also increase, even when the proportions are unchanged, assuming that fuel has not started to become a limitation to the lifespan of a fire. The variation in proportional fire weather potentials becoming realized among some of the Canadian Ecozones may reflect fire ignition limitations.

When is fire weather extreme enough for active fire spread in Canada? The answer is quite clear: fire could spread at average fire-conducive weather conditions, as long as it is hot, dry and windy; however, the more extreme the conditions, the greater the likelihood for fire to spread. The thresholds proposed in this study should not be viewed as switch values. They represent the fire weather conditions when most of the area burned based on the fire and fire weather records in about the past 20 years. Fire could still spread quickly with a lower fire weather condition but is comparatively less likely. In addition, we do recommend that practitioners consider the range of fire thresholds, as proposed in [25], and use these numbers as references to identify whether or not a spread day may occur.

## 5. Conclusion

This study identified the critical fire weather conditions for active fire growth in Canada at national and Ecozone levels. With these thresholds, this study demonstrated that the median fire weather conditions quantified by the FWI System variables are sufficient for active fire growth, and on average about 22 to 30% of such days may be realized in real fire spread at various Canadian Ecozones. The results of this study improved the accuracy of spread day thresholds recommended in the previous studies. This study also demonstrated the importance of potential fire-conducive conditions to fire activity, which could be used to project future fire activity with the changing climate.

## Data Availability

Data are publicly available and can be accessed through Canadian Forest Service datamart (https://cwfis.cfs.nrcan.gc.ca/datamart). Supplementary material is available online [40].
